# DO‐SRS imaging of diet regulated metabolic activities in Drosophila during aging processes

**DOI:** 10.1111/acel.13586

**Published:** 2022-03-07

**Authors:** Yajuan Li, Wenxu Zhang, Anthony A. Fung, Lingyan Shi

**Affiliations:** ^1^ Department of Bioengineering University of California San Diego La Jolla California USA

**Keywords:** aging, diet, DO‐SRS, Drosophila, fatbody, heavy water, metabolism, stimulated Raman scattering

## Abstract

Lipid metabolism plays crucial roles during aging processes, but how it is regulated by diets and how it interplays with aging still remain unclear. We proposed a new optical imaging platform by integrating heavy water (D_2_O) probing with stimulated Raman scattering (DO‐SRS) microscopy, for the first time, to directly visualize and quantify lipid metabolism regulated by different diets and insulin signaling pathway in Drosophila fat body during aging. We found that calorie restriction, low protein diet, and (moderately) high protein and high sucrose diets enhanced lipid turnover in flies at all ages, while (moderately) high fructose and glucose diets only promoted lipid turnover in aged flies. The measured lipid turnover enhancements under diverse diets were due to different mechanisms. High protein diet shortened the lifespan while all other diets extended the lifespan. Downregulating the insulin signaling pathway enhanced lipid turnover, which is likely related to lifespan increase, while upregulating insulin signaling pathway decreased lipid turnover that would shorten the lifespan. Our study offers the first approach to directly visualize spatiotemporal alterations of lipid turnover in aging Drosophila in situ, for a better understanding of the interconnections between lipid metabolism, diets, and aging.

## Graphical abstract

We directly visualized and quantified lipid metabolism regulated by different diets and insulin signaling pathway, respectively, in situ in Drosophila fat body during aging. We found that calorie restriction, low protein diet, and (moderately) high protein and high sucrose diets enhanced lipid turnover in flies at all ages, while (moderately) high fructose and glucose diets only promoted lipid turnover in aged flies.

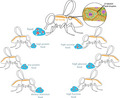

**Abbreviations**
C‐D: carbon‐deuterium
C‐H: carbon‐hydrogen
CR: calorie restriction
D_2_O: deuterium oxide, heavy water
DO‐SRS: deuterium oxide probed stimulated Raman scattering
FA: fatty acid
HF: high fructose
HG: high glucose
HP: high protein
HS: high sucrose
LD: lipid droplet
LP: low protein
InR: insulin receptor
ST: standard food
SRS: stimulated Raman scattering

## 1 INTRODUCTION

Lipids play important roles in the body. They not only constitute cell membranes, but also function as energy storage and supplier, and as messengers in signaling pathways (Meer et al., 2008). Lipid metabolism is a key nutrition‐related signaling network that regulates responses to calorie restriction and dietary composition. Emerging studies have shown that lipid metabolism plays an important role in the aging process (reviewed by Johnson & Stolzing, 2019), in which the lifespan in animal models such as *C. elegans*, fruit flies, and rodents was extended by different types of lipid‐related intervention, including dietary manipulation. Disruption of lipid homeostasis by excess calorie intake leads to obesity, which is a major risk factor for diabetes, cardiovascular disease, stroke, and incidence of cancer (Bergström et al., 2001a, 2001b; Kloska et al., 2020; Parhofer, 2015; Peck & Schulze, 2019; Renehan et al., 2008). On the contrary, calorie restriction (CR) extends longevity and delays the occurrence and progression of age‐associated diseases in organisms ranging from yeast, various invertebrates, to rodents and primates (Balasubramanian et al., 2017; Mair et al., 2004). Diverse dietary composition including protein, fat, and carbohydrates also impact the life span and metabolic health in animals such as Drosophila and mice. However, the regulation of dietary composition on lipid metabolism and, consequently, the mechanisms underlying aging process in response to diet‐manipulated lipid metabolism are still not fully understood.

A variety of techniques have been employed for lipidomics studies. Mass spectrometry (MS)‐based techniques are most widely used, including gas chromatography (GC)‐MS, liquid chromatography (LC)‐MS, and matrix‐assisted laser desorption/ionization (MALDI)‐MS. However, GC‐MS and LC‐MS are destructive to live tissues and can hardly obtain the spatial information of lipids in situ, while MALDI imaging can detect spatial distribution of lipids but it has relatively low spatial resolution (Bowman et al., 2020) and limited imaging depth (<15 µm) (Murphy et al., 2009). Other techniques provide direct means to image lipid distributions inside tissues but also with some limitations, such as low spatial resolution (magnetic resonance imaging), loss of spatial information (nuclear magnetic resonance spectroscopic imaging), and requirement of bulky fluorescent dyes that will disturb the native metabolic activities (fluorescence microscopy). Raman spectroscopy/microscopy has emerged and evolved quickly as a powerful modality for molecular imaging and has displayed many advantages including high resolution (subcellular), non‐invasiveness, high chemical specificity, and multiplex imaging capability (Daudon & Bazin, 2016; Ember et al., 2017; Ghita et al., 2012; Kong et al., 2013). Incorporated with different probes, Raman imaging can be applied for visualizing a variety of biomolecules including lipids, protein, and DNA, in living cells and organisms (Berry et al., 2015; Henk‐Jan van Manen & Otto, 2008; Shi et al., 2018; Wang et al., 2020; Zhang et al., 2019). Most recently, we have developed a new imaging platform that integrates heavy water (D_2_O) probing with stimulated Raman scattering (DO‐SRS) microscopy. Similar as water, D_2_O can diffuse freely into the cells and deuterium (D) from D_2_O is incorporated into newly synthesized biomolecules such as lipids through de novo lipogenesis to form carbon‐deuterium (C–D) bonds, which enables us to visualize metabolic dynamic in organisms in situ. Using this platform, we visualized metabolic dynamics of multiple biomolecules simultaneously in living animals in situ (Shi et al., 2018).

Here, for the first time, we applied D_2_O‐probed Raman and DO‐SRS microscopy to visualize lipid metabolic activities in the fat body of Drosophila melanogaster during aging. We examined lipid turnover rate in lipid droplets (LDs) of Drosophila that were regulated by diverse dietary compositions and insulin signaling pathway, respectively. Recent study (Arner et al., 2019) investigated lipid turnover (the storage and removal of lipids) changes in human and discovered decrease in lipid removal rate but no change in lipid uptake rate with aging, resulting in a 20% weight gain over 13‐year period, while a therapeutic weight loss over 5‐year period was not due to lipid removal but the decrease of lipid storage. In our study, by quantitating lipid turnover rate with the ratio of C–D signal (newly synthesized lipids) to C–H signal (old lipids), we investigated the impacts of diet composition on lipid metabolism and life span, as well as insulin pathway on lipid metabolism, for a better understanding of the roles lipid metabolism plays in aging and life span.

## 2 RESULTS

### 2.1 DO‐SRS imaging of diet‐regulated Drosophila lifespan and lipid metabolic activity

We first investigated the impacts of dietary compositions on female Drosophila lifespan and lipid metabolic dynamics in the fat body. Studies have shown that supplementation of dietary fatty acids (FAs) can extend the lifespan in *C. elegans* by regulating lipid metabolism (Papsdorf & Brunet, 2019).

Previous lipidomic study (Carvalho et al., 2012) on *Drosophila* found metabolized dietary lipids can affect the composition of membrane lipidomes of tissues, such as gut and imaginal disks. In contrast, triacylglycerol (TAG) species in the fat body are much less influenced by the FA composition in the diet, even though the fat body stores much more TAG when larvae are fed lipid‐rich food. The fat body can even accumulate normal TAG stored in a lipid‐free diet. This indicates that the fat body is not a passive depot for storage of dietary lipids but its endogenous synthesis supplies a large fraction of fat body TAG (Carvalho et al., 2012).

We used a simplified recipe that mainly contains 100 g yeast, 50 g sucrose, 8 g cornmeal, and 10 g agar as the standard diet (Table S1) (Bass et al., 2007). The yeast is the source for protein and lipids, and sucrose is the source for carbohydrates. For high glucose or fructose diets, sucrose was replaced with glucose or fructose, respectively. The dietary balance of protein to carbohydrate (P:C) ratio has been shown the predominant determinant for the lifespan (Lee, 2015). For example, increase in the yeast to sucrose ratio can decrease the life span of Drosophila (Krittika & Yadav, 2020; Lee, 2015; Zid et al., 2009).

**FIGURE 1:**
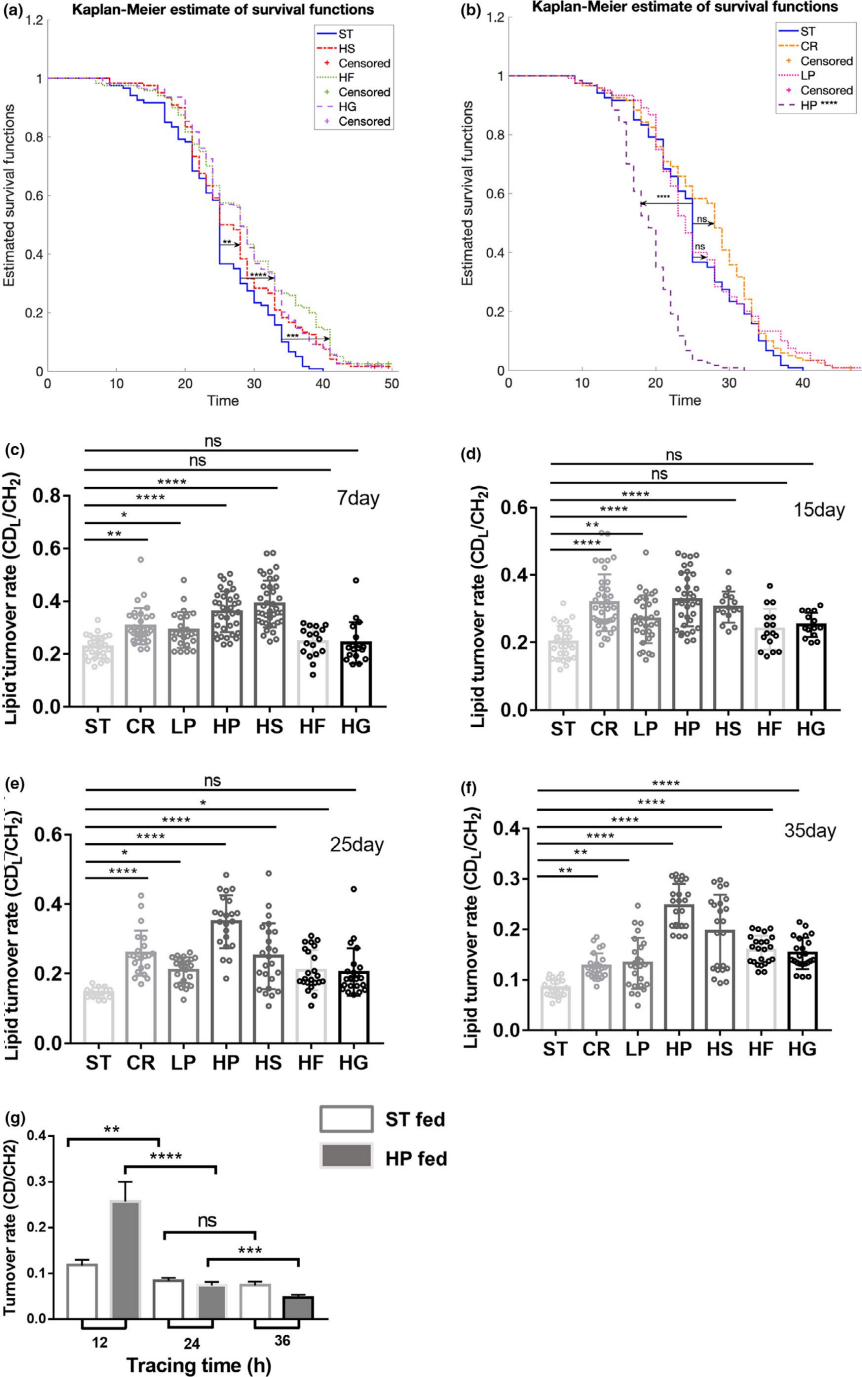
Diet‐regulated lifespan and lipid metabolic activity in flies with aging. The lifespan of Drosophila on high sugar diets (a), high/low protein diets and calorie restriction (b) are compared. The Kaplan–Meier estimator was used for survival analysis. *n* = 120, separated to 4 cohorts for each dietary condition. (c–f) Quantification of diet‐manipulated lipid turnover in flies at varied ages. (g) Metabolic turnover in flies on starvation resistance test. Flies previously on high protein diet had a significantly high turnover in the first 12 h or tracing but it greatly declined at 36 h, showing a significantly high catabolism in flies previously on high protein diet. Results are presented as Mean ± SD (*n* = 25, from 3 to 5 different individuals in each group). Statistical significance was determined by using one‐way ANOVA. *, *p* < 0.05; **, *p* < 0.01; ****, *p* < 0.0001; ns, non‐significant difference

We first examined the lifespan of Drosophila manipulated by diverse dietary compositions. We fed adult flies 20% D_2_O‐labeled foods containing different proportions of sugar and yeast. In high sugar (2× sugar) diets, we examined influences of high glucose, high fructose, and high sucrose, respectively. We discovered that they all extended the flies’ ultimate lifespan to 50 ± 1 days compared with 41 ± 1 days of flies on normal diet, while high glucose and high fructose diets both led to longer median lifespan (29 ± 1 days) than high sucrose (27.5 ± 1 days) (Figure 1a), suggesting *Drosophila* used monosaccharides and disaccharides in different ways. High protein diet (2× protein) was found to shorten the ultimate lifespan to 33 ± 1 days and the median lifespan to 19.6 ± 1 days, respectively (Figure 1b). Low protein diet (1/2 × protein) increased the ultimate life span to 49 days but did not change the median life span (Figure 1b). Calorie restriction diet (1/2 × sugar, 1/2 × protein) also extended the ultimate lifespan to 49 days, but only increased the median lifespan from 25 to 29.8 ± 1 days (Figure 1b). Different from carbohydrate‐rich diets that always resulted in significantly higher fat storage, diets composed largely of calories from yeast produced lean flies, and leanness was preserved in these yeast‐rich diets even in cases where total calorie intake was substantial (Skorupa et al., 2008). For a given level of estimated calories consumed, the type of calorie intake influences the lifespan (Skorupa et al., 2008). Our study demonstrates that high carbohydrate and low protein diets extended Drosophila life span, while high protein diets shortened the lifespan (Piper et al., 2011; Simpson et al., 2017).

To elucidate the interconnections between lipid metabolism, aging, and diet manipulation, we further tracked and quantitated lipid (and other biomolecules’) metabolic activity regulated by diets in Drosophila during development and aging, using DO‐Raman and DO‐SRS imaging. Female Drosophilae at 2, 10, 20, and 30 days after eclosion, respectively, were transferred from standard diet to 20% D_2_O‐labeled foods that containing diverse dietary compositions (high sugar, high protein, etc.) and fed for 5 days. Then the fat body was dissected and lipid metabolism was measured by Raman and SRS imaging, respectively. Raman spectra showed no evident difference among 7‐ and 15‐day flies posteclosion on diverse diets (Figure S1a,b), but the differences among 25‐day flies are obvious, at 1301 cm^−1^ (lipids and protein amide III), 1580 cm^−1^ (nucleic acids), and 2140 cm^−1^ (CD band), and even more evident among 35‐day‐old flies (Figure S1c,d). The differences of these peaks between old (35‐day) and young (7‐day) flies are also significant, indicating dramatic alterations of these biomolecules during aging.

We then quantified lipid turnover rate influenced by dietary composition at different groups (Figure 1c–f). The lipid turnover rate significantly declined in aging flies on standard diet. Compared with standard diet, high sucrose, high protein, calorie restriction, and low protein diets significantly enhanced lipid turnover rates in flies at all ages from 7 to 35 days, but high fructose and high glucose did not significantly change lipid turnover until at Days 25 and 35, respectively (Figure 1c–f). The different effects between sucrose and glucose/fructose on lipid turnover in young flies (7–25 days) suggest that calorie may not be the only factor that regulates lipid metabolism, and monosaccharides and disaccharides are metabolized differently in the body, which is likely associated with insulin resistance. In aged flies (35‐day), the promotion of high sugar diets on lipid synthesis became dominant, as all 3 high diets led to significant increases of lipid turnover. Calorie restriction and low protein diets reduced insulin resistance and increased insulin sensitivity that stimulated more lipid synthesis (Toyoshima et al., 2010). The temporal changes of lipid turnover rate on each individual diet are also shown in Figure S1. Among all diets, high protein diet played the most significant role in enhancing lipid turnover rates at almost all ages (only next to high sucrose diet at 7‐day); however, it also shortened the lifespan (Figure 1b). This is due to the insulinotropic effect of dietary protein that elevates insulin secretion, promotes lipid excretion, and slows lipid absorption and synthesis (El Khoury & Anderson, 2013; Rietman et al., 2014), which displays as increase in lipid turnover rate, while upregulation of insulin signaling pathway shortened the life span (Chung, 2021).

To test whether lipid catabolism was greatly increased along with increased anabolism in Drosophila on high protein diet, we performed starvation resistance test on Drosophila subjected to high protein diet (test group) and standard test (control group), respectively. We fed flies 20% D_2_O‐labeled high protein diet and standard diet for 5 days, transferred the flies to vial, and gave them access to water but not to food. We observed that all flies that were previously on high protein diet died rapidly within 48 h of starvation, while flies previously on standard diet died within 120 h of starvation. This showed that greatly enhanced lipid excretion in flies on high protein diet caused the quick death of flies once they were on starvation. We further measured C‐D turnover rate during the starvation resistance test. In flies previously on high protein diet, we measured significantly higher C‐D turnover rate than the control at 12 h of starvation, but the turnover rate dramatically decreased to the same level as the control at 24‐h, and a much lower level at 36‐h after starvation (Figure 1g). This dramatic decline of CD turnover rate was evidently due to the high lipid catabolism in flies on high protein diet.

In addition to lipid metabolism, we also quantitated diet influence on retinoids, protein, and unsaturated FAs relatively to the CH_2_ lipids stretching at 2850 cm^−1^ (Figure S2). We observed these biomolecules all changed in the same trends as in the flies on standard diet, that is, significant increases of retinoids and unsaturated FAs but dramatic drop of protein with aging, suggesting the influence of dietary composition was not obvious.

### 2.2 DO‐SRS imaging of spatiotemporal alterations of lipid droplets by diet

**FIGURE 2:**
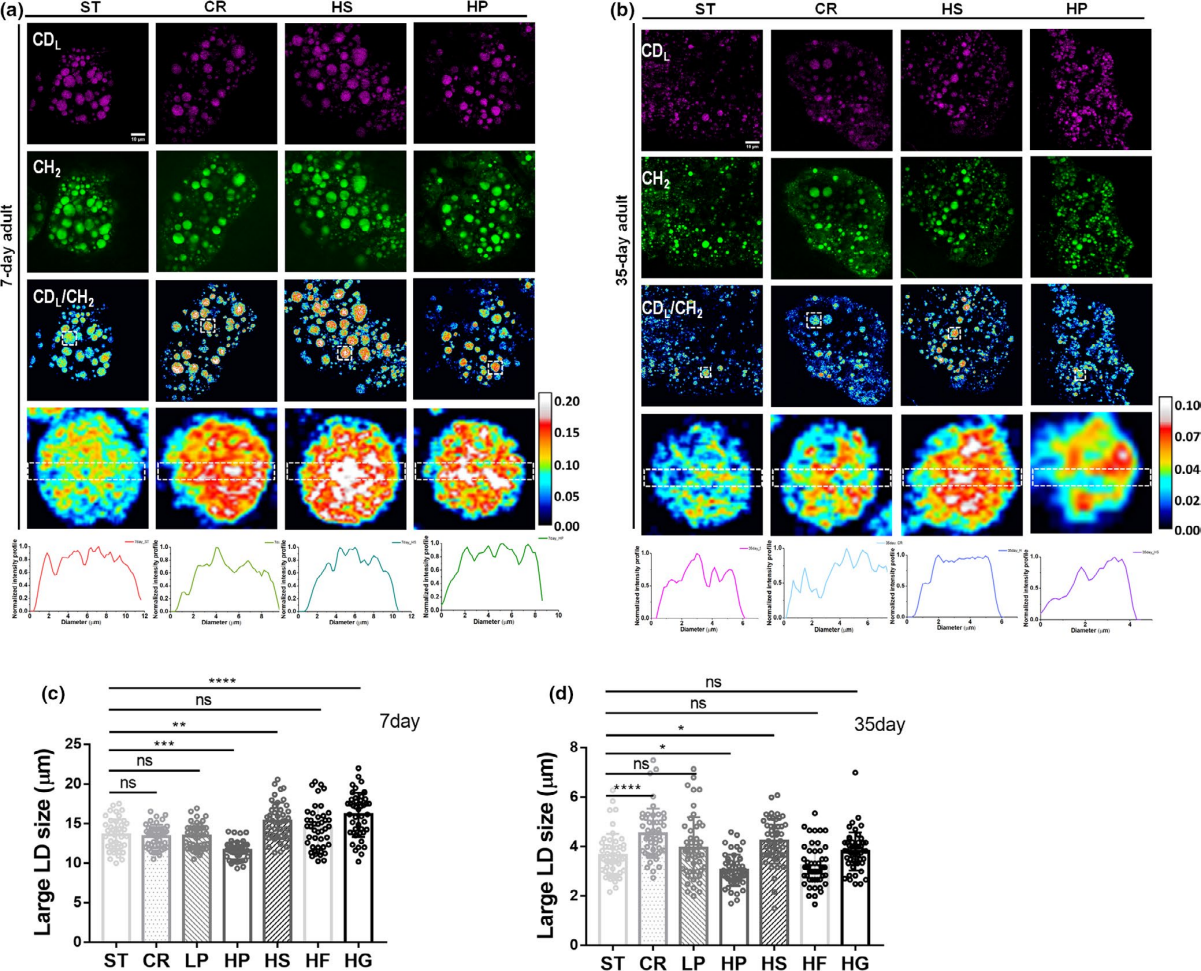
SRS imaging of diet‐regulated lipid metabolic activity in young (a) and old (b) flies. The lipid turnover profiles inside single LDs were also plotted as curves. The average sizes of large LDs were compared in young (c) and old (d) flies manipulated by diverse diets. ST, standard diet; CR, calorie restriction; LP, low protein; HP, high protein; HS, high sucrose; HF, high fructose; and HG, high glucose. Results are presented as Mean ± SD (*n* = 50, from 5 different individuals in each group). Statistical significance was determined by using one‐way ANOVA. *, *p* < 0.05; **, *p* < 0.01; ***, *p* < 0.001; ****, *p* < 0.0001; ns, non‐significant difference

Using DO‐SRS imaging, we were able to examine lipid turnover and mapping their distribution inside individual LDs of Drosophila manipulated by calorie restriction, high sucrose, and high protein diets, respectively (Figure 2a,b). Ratiometric images of newly synthesized lipids at 2140 cm^‐1^ to the peak at 2850 cm^−1^ (CH_2_ stretching of lipids) showed significant lipid turnover in all diet manipulations compared with standard diet in both young and aged flies. The newly synthesized lipids were evenly distributed inside the LDs in young flies (Figure 2a) but localized in particular regions of the LDs in aged flies (Figure 2b). Compared with young flies, lipid turnover rates in aged flies under all diets were dramatically reduced. In addition, from SRS images we can visualize that there are more large LDs than small LDs in young flies, but much more small LDs in aged flies. Since LD size is considered to be associated with lipid storage capability, reduced LD size in aged flies indicates the reduction of lipid storage capability in flies during aging.

We measured significant reductions of large LD size in aging flies (unpublished data), which showed the loss of lipid storage capacity. To examine the influences of diverse diets on large LD size during aging, we used DO‐SRS imaging to directly visualize metabolism in LDs. We found the overall averaged diameter of large LD decreased from ~14 µm in 7‐day young flies (Figures 2c, S3b,d) to ~4 µm in aged flies (Figures 2d, S3b,d). Under diverse diet manipulations in both young and old flies, LD sizes increased by high sugar diets, significantly or moderately (high fructose), but decreased dramatically by high protein diet (Figures 2c,d, S3a,c). This further confirms that high protein diet accelerated lipid excretion and slowed lipid storage, causing the shrink of LDs, and the elevated lipid turnover was due to the reduction of total lipids. High sugar diets maintained or promoted lipid storage in flies, shown as dramatically or moderately changed LD size and elevated lipid turnover due to increased lipid storage. Taken together our observations of flies’ lifespan regulation by different diets (Figure 1a,b), our study showed that aged flies with longer lifespan (as regulated by high sugar diets and low protein diet) tend to contain more large LDs for lipid storage in the fat body. On the contrary, aged flies with more small LDs in the fat body had a shortened lifespan due to the loss of lipid storage, as manipulated by high protein diet.

### 2.3 Quantifying lipid metabolism in aging Drosophila mutants

The insulin/IGF‐1 signaling (IIS) pathway plays an essential role in regulating the lifespan and the metabolic homeostasis of glucose and lipids (Nässel et al., 2015). Genetic inactivation of the IIS pathway in *Drosophila* and *C. elegans* has been shown to extend their lifespans (Broughton et al., 2005; Clancy et al., 2001; Kenyon et al., 1993; Giannakou et al., 2004; Nässel et al., 2015). The phosphoinositide 3‐kinase (PI3K) is a key IIS effector molecule. Inhibiting the insulin receptor (InR)/PI3K signaling pathway in Drosophila has been shown to phenocopy the effects of starvation, while activating it caused starvation sensitivity in the organism, which shows InR/PI3K signaling pathway plays an essential role in controlling Drosophila growth and in regulating cellular metabolism with diet (Britton et al., 2002). To further identify the mechanisms interconnecting metabolism and aging by InR/PI3K pathway, we applied DO‐SRS to visualize and quantify lipid metabolic activity in Drosophila genetic mutants.

**FIGURE 3:**
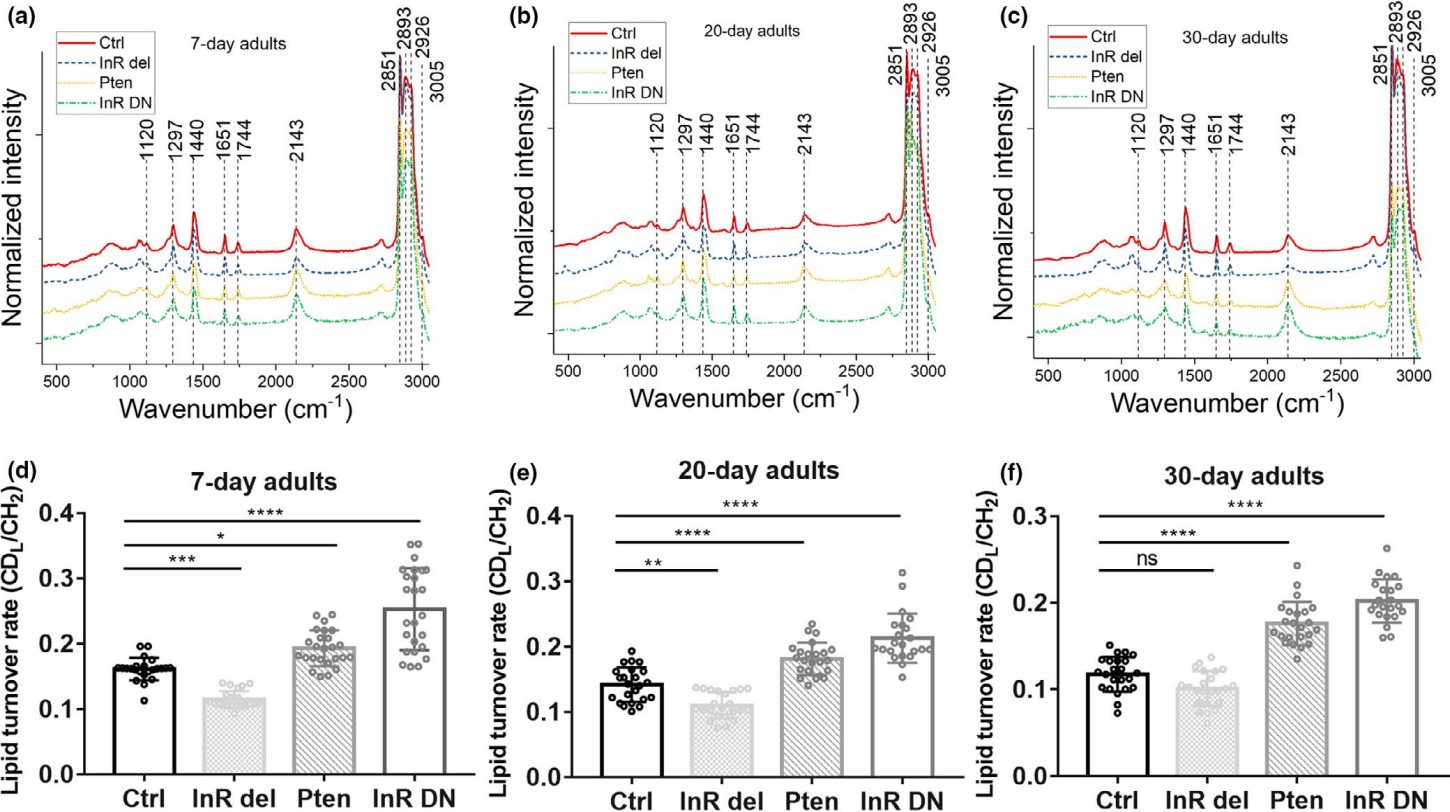
Lipid turnover rate in mutant flies with aging. (a–c) Raman spectra of fat body in control and mutant flies at 7‐, 20‐, and 30‐day posteclosion. Before imaging, flies were fed 20% D_2_O‐labeled food for 5 days. Peaks at 2143 cm^−1^ represent the newly synthesized lipids. (d‐f) Quantification of lipid turnover rates in flies at different ages. Ctrl, control *ppl*‐*G4*/+ fly; *InR del*, *ppl*‐*Gal4*>*UAS*‐*InR del* mutant fly; Pten, *ppl*‐*Gal4*>*UAS*‐*Pten* mutant fly; InR DN, *ppl*‐*Gal4*>*UAS*‐*InR DN* mutant fly. Results are presented as Mean ± SD (*n* = 25, from 3 different individuals in each group). Statistical significance was determined by using one‐way ANOVA. *, *p* < 0.05; **, *p* < 0.01; ***, *p* < 0.001; ****, *p* < 0.0001; ns, non‐significant difference

We used the *ppl*‐*Gal4*, a specific *Gal4* driver in the fat body of Drosophila, to drive *UAS*‐*Pten* (a lipid phosphatase counteracting PI3K enzymatic activity) (Nowak et al., 2013) and *InR*‐*DN* (the dominant‐negative form of InR) expression, and *InR del* (the catalytic constitutive active form of InR) flies 2‐, 15‐, and 25‐day posteclosion that were fed D_2_O‐labeled standard food for 5 days (i.e., 7‐, 20‐, and 30‐day posteclosion). We then applied D_2_O‐probed Raman microscopy to examine metabolic activity in Drosophila at different age groups (Figure 3a–c). Evidently higher peaks at 2143 cm^−1^ could be observed in *ppl*‐*Gal4*>*UAS*‐*Pten* and *ppl*‐*Gal4*>*UAS*‐*InR DN* flies compared with control (Figure 3a–c). Quantification of the lipid turnover rate showed that in both young and old mutant flies, upregulation of the InR/PI3K signaling pathway as in *ppl*‐*Gal4*>*UAS*‐*InR del* flies reduced lipid metabolism (Figure 3d–f), while inhibiting or inactivating the InR/PI3K signaling pathway in *ppl*‐*Gal4*>*UAS*‐*Pten* and *ppl*‐*Gal4*>*UAS*‐*InR DN* flies significantly increased lipid turnover in flies at all ages (Figure 3d–f). Further comparing lipid metabolism in each fly mutant during aging revealed that genetic downregulation of InR/PI3K pathway would maintain the relatively high lipid metabolism in flies (*ppl*‐*Gal4*>*UAS*‐*Pten*) with aging (Figure S4c), but upregulation (*ppl*‐*Gal4*>*UAS*‐*InR del*) or inactivation (*ppl*‐*Gal4*>*UAS*‐*InR DN*) of the pathway led to significant decreases of lipid metabolism in aged flies (Figure S4b,d), similarly as in the control (Figure S4a). A recent study on mouse adipose tissue and fly fat body showed that downregulation of InR/PI3K pathway increased lipid metabolism as a conserved effect (Bettedi et al., 2020), and downregulation by genetic modulation of the IIS pathway in invertebrates has been associated with prolonged lifespan (Tatar et al., 2003). Our results also revealed the increased lipid metabolism as a conserved effect of downregulating InR/PI3K pathway, as in *ppl*‐*Gal4*>*UAS*‐*Pten* flies.

### 2.4 SRS imaging of spatiotemporal alterations of lipid metabolism in aging Drosophila mutants

**FIGURE 4:**
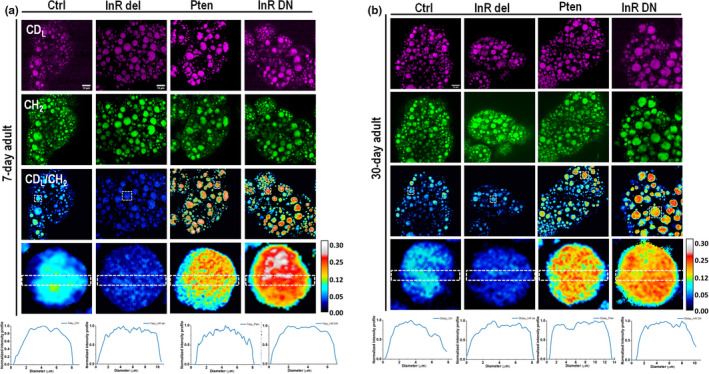
DO‐SRS imaging of lipid metabolism in the fat body of mutant flies at (a) 7‐day and (b) 30‐day posteclosion. The ratiometric images (CD_L_/CH_2_) display the ratio of CD signal at 2143 cm^−1^ (newly synthesized lipids) to the signal at 2850 cm^−1^ (CH_2_ stretching of lipids). The genetic downregulation of InR/PI3K pathway (Pten, *ppl*‐*Gal4*>*UAS*‐*Pten*; InR DN, *ppl*‐*Gal4*>*UAS*‐*InR DN*) showed higher lipid metabolism than control, and upregulation of InR/PI3K pathway (InR del, *ppl*‐*Gal4*>*UAS*‐*InR del*) led to reduced lipid metabolism in both young and old flies

We further applied DO‐SRS imaging to directly visualize lipid metabolism and map distribution of newly synthesized lipids in the fat body. In both young and old flies, SRS images (Figure 4) demonstrated much stronger C‐D signals in *ppl*‐*Gal4*>*UAS*‐*Pten* and *ppl*‐*Gal4*>*UAS*‐*InR DN* mutant flies than control, which indicates more newly synthesized lipids in the fat body, and weaker C‐D signals in *ppl*‐*Gal4*>*UAS*‐*InR del* due to less lipids synthesized. The ratiometric images of newly synthesized lipids at 2143 cm^−1^ to the CH_2_ stretching of lipids (peak at 2850 cm^−1^) showed that the lipid turnovers were evenly distributed inside the LDs in all flies. Comparison of 7‐day (Figure 4a) and 30‐day (Figure 4b) flies showed no dramatic reduction of lipid turnover in old *ppl*‐*Gal4*>*UAS*‐*InR Pten* (*Pten*) flies, but in other mutants and control flies the lipid turnover was evidently reduced, as shown by the quantification results in Figure S4.

### 2.5 Quantification of fatty acids storage in aging Drosophila mutants

**FIGURE 5:**
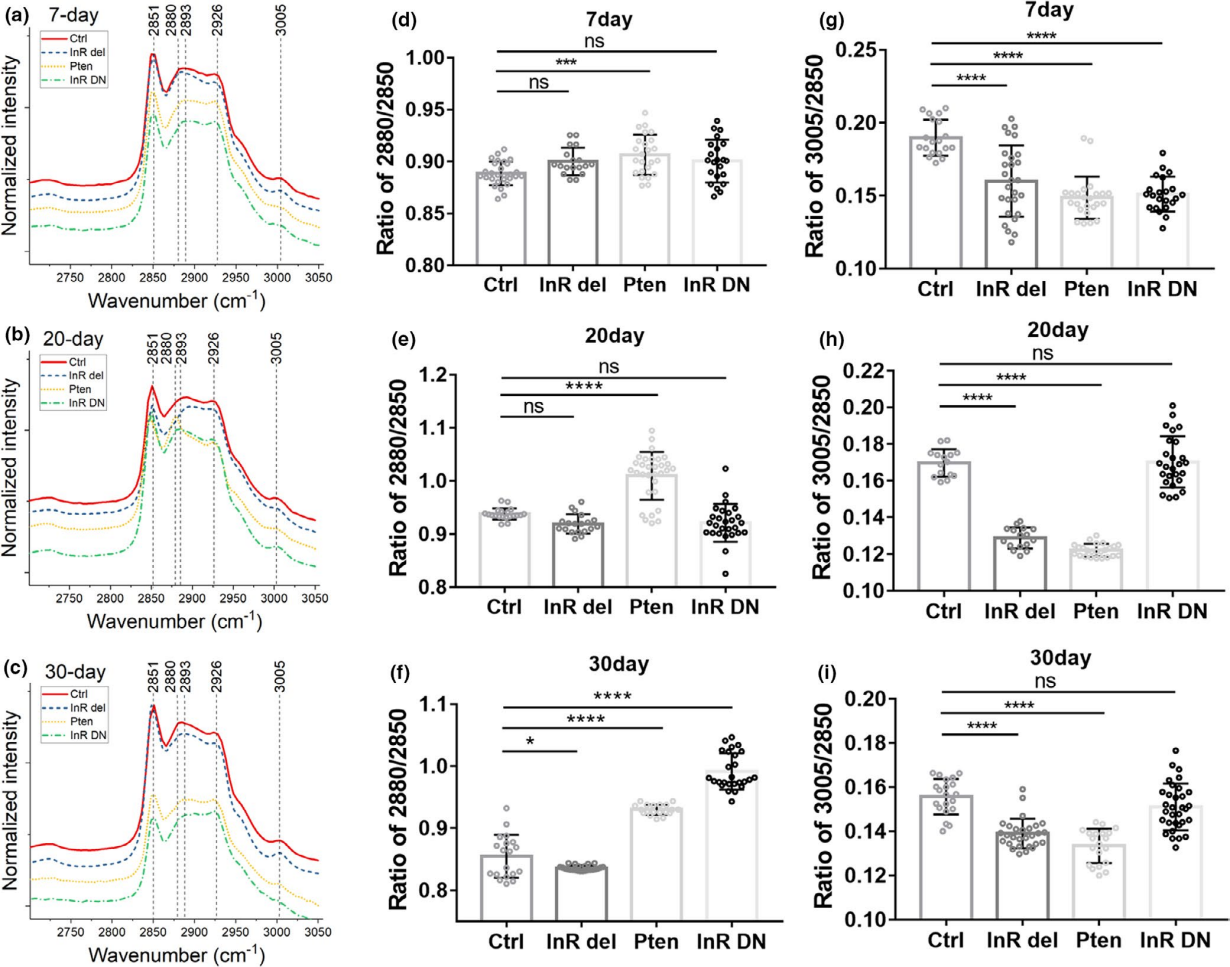
Changes of saturated and unsaturated FAs storage in mutant flies during aging. (a)–(c) Raman spectra at the C–H stretching region showed changes of peaks at 2880 cm^−1^ (saturated FAs) and 3005 cm^−1^ (unsaturated FAs) at 7‐, 20‐, and 30‐day, respectively. (d)–(f) Quantification of saturated FAs at 2880 cm^−1^ contents relative to the CH_2_ lipids stretching at 2850 cm^−1^ in mutant flies at 7‐, 20, and 30‐day posteclosion. (g)–(i) Quantification of unsaturated FAs at 3005 cm^−1^ content relative to peak at 2850 cm^−1^ in mutant flies at different ages. Results are presented as Mean ± SD (*n* = 25, from 3 different individuals in each group). Statistical significance was determined by using one‐way ANOVA. *, *p* < 0.05; ***, *p* < 0.001; ****, *p* < 0.0001; ns, non‐significant difference

Excess storage of saturated fat in white adipose tissue causes proinflammatory response within the tissue. The proinflammation and saturated FAs released into the bloodstream will lead to insulin resistance in the body (Funaki, 2009). With Drosophila insulin pathway mutants, we used D_2_O‐probed Raman microscopy to examine and quantitate the relative storages of saturated and unsaturated FAs in the fat body of aging flies. In addition to the changes of C‐D signal (newly synthesized lipids) at 2143 cm^−1^, the Raman spectra obtained previously (in Figure 3) also reveal changes of saturated and unsaturated FAs at 2880 cm^−1^ and 3005 cm^−1^, respectively (Figure 5a–c, zoomed‐in spectra of Figure 3). Quantification results (Figure 5d–f) show that upregulating the insulin pathway (as in *InR del*) reduced saturated FAs storage in old flies, while inhibiting the insulin pathway (*Pten*) significantly increased saturated FAs storage in flies at all ages, and inactivating insulin pathway (*InR DN*) only significantly elevated the storage in old flies. Unsaturated FA contents were significantly reduced in all young mutant flies (Figure 5g), suggesting any disturbance to the insulin pathway would lead to the decrease of the unsaturated FAs. The storage of unsaturated FAs in *ppl*‐*Gal4*>*UAS*‐*InR DN* flies (inactivating insulin pathway) was restored to normal at both 20‐ and 30‐day posteclosion (Figure 5h,i), but still significantly lower than control in *ppl*‐*Gal4*>*UAS*‐*InR del* and *ppl*‐*Gal4*>*UAS*‐*Pten* flies. Here, we successfully employed a new approach to investigate the correlation of saturated FAs and insulin signaling pathway by using D_2_O‐probed Raman and DO‐SRS imaging.

## 3 DISCUSSION

For the first time, we established a Drosophila model for studying metabolic activities regulated by diverse diet compositions and insulin pathway, respectively, using D_2_O probed SRS imaging. This imaging platform allows us to directly visualize and quantitate the spatiotemporal changes of lipids as well as other biomolecules metabolic turnover inside Drosophila fat body cells in situ. We examined the interconnections between lipid metabolism, dietary composition, insulin pathway, and life span.

Recent studies have shown that lipid metabolism not only is associated with aging and age‐related diseases, but also actively participates in the regulation of development and aging (Chung, 2021; Johnson & Stolzing, 2019). Lipid‐related dietary, genetic, and pharmaceutical interventions are able to extend or shorten the life span. We found high protein diet dramatically increased lipid catabolism and slowed lipid storage in flies, as shown by the reduction of LD size and the starvation resistance test that shortened the life span. High sugar (twofold, sucrose, glucose, and fructose) diets promoted lipid storage (enlarged LDs) that extended flies’ life span. Although we measured elevated lipid turnover rates in both high protein and high sugar diets, they were due to different mechanisms. Lipid turnover increase in high protein diet was due to the dramatic excretion of lipids, while in high sugar diets it was due to enhanced lipid storage. Previous studies have shown that 10‐fold sugar diets shortened the life span of flies (Musselman et al., 2011; van Dam et al., 2020), but our study showed lifespan extension in flies on twofold high sugar diets, which may be due to moderate increase of sugar contents in our study compared with 10‐fold increase. To assess the effect of diverse sugar content on lifespan, we fed flies twofold, fourfold, and 10‐fold sucrose diets, respectively. We found that 10‐fold sugar diet significantly shortened the median lifespan from 49 ± 1 days to 27 ± 1 days, fourfold sugar diet slightly reduced the lifespan to 47 ± 1 days, but twofold sugar diet extended the lifespan to 52 ± 1 days (Figure S5). Our results demonstrated that different concentrations of sugar diets would play opposite roles in regulating the lifespan. Moderately high sugar will extend the lifespan but excessively high sugar will significantly shorten the lifespan. Nevertheless, the underlying mechanisms need further investigation.

We examined lipid turnover regulated by insulin signaling pathway. Lipid turnover declined when the InR/PI3K insulin pathway was upregulated as in *InR del* flies, and significantly increased when the pathway was downregulated (in *Pten* and *InR DN* flies). On the contrary, decreased insulin signaling pathway extends the life span (Chung, 2021). Our study showed that enhancement of lipid turnover rate (not due to increased lipid excretion as by high protein diet) would prolong the lifespan, which is associated with the downregulation of the insulin pathway.

In summary, our study presents a new approach of optical imaging that can directly visualize and quantify spatiotemporal alterations of lipid turnover in Drosophila fat body in situ at subcellular scale, toward a better understanding of the impacts of dietary compositions and insulin pathway on metabolism and longevity in flies. The new optical imaging platform shows broad applications for studying metabolism, aging, and age‐related diseases in live animals in situ.

## 4 MATERIALS AND METHODS

### 4.1 Fly stocks

Wild type (*w^1118^* stock #5905) and insulin signaling pathway‐related transgenic flies (*UAS*‐*InR*.*del*, #8248; *ppl*‐*Gal4*, #58768; *UAS*‐*InR*.*DN*, #8253; *UAS*‐*Pten*, #82170) were originally obtained from the Bloomington Stock Center and have been maintained in the laboratory for several generations.

### 4.2 *Drosophila* lifespan and diet manipulation analysis

The *w^1118^* parents were raised in vials containing standard food. To standardize the effects of parental age on offspring fitness, parents of experimental flies were of the same age (4–5 days and reared at a constant density for at least two generations). To synchronize larval development, we allowed flies to lay eggs on yeast apple juice plates for 1 h, discarded the first batch of embryos, and then collected for another 1.5 h. Groups of 20–25 embryos were put into vials containing standard food and allowed to develop until pupae eclosion. Groups of adult progenies emerged from puparium within 24 h were transferred to fresh bottles and allowed to mate for 2 days.

For the diet‐regulation experiments, 2 days aged *w^1118^* females were separated under CO_2_ anesthesia and randomly allocated to different media (approximately 20–30 flies per vial) using 4 cohorts for each of 7 diets: standard food (ST), calorie restriction (CR), low protein (LP), high protein (HP), high sucrose (HS), high fructose (HF), and high glucose (HG). For the detail information of the food recipe, see Table S1. All eclosures were maintained at 25°C in a controlled light (12/12‐h light/dark cycle) and humidity (>70%) environment. Flies were scored for survival daily and provided with fresh medium every 2 days. To minimize any density effects on mortality, two vials with cohorts were merged when the density of flies reached five or fewer individuals. Eclosures were placed randomly in the incubator, and positions were rotated after each transfer to minimize the effects of microclimate. Three independent trials with about 100 flies per diet were performed. The life span curves for each diet represent cumulative survival for about 300 flies. For each diet treatment, median and maximum lifespan were calculated as half of deaths of the whole population and the longest lived 10% of individuals, respectively.

### 4.3 D_2_O‐labeling experiments

The metabolic activity changes of wild type flies at different ages were labeled by transferring the 2‐day, 10‐day, 20‐day, and 30‐day female adult flies to the 20% D2O‐labeled corresponding food conditions for 5 days, then the 7‐day, 15‐day, 25‐day and 35‐day aged flies were sacrificed, and fat body was dissected and subjected to Raman measurements and SRS imaging.

For the insulin signaling‐related gene knocking‐down experiments, groups of 15 *ppl*‐*Gal4* virgin females were collected to cross with 15 *UAS*‐*InR*.*del*, *UAS*‐*InR*.*DN*, and *UAS*‐*Pten* males, respectively. All the crosses were maintained at 21°C on the standard food to allow the normal embryo and larvae development. 20 newly enclosed progenies (10 males and 10 females) with correct genotype were collected and transferred to the fresh food and raised in 25°C to allow the *Gal4*‐*UAS* system function well. After 2 days maturation and mating, the female flies were separated and subjected to 5‐day 20% D2O labeling experiments; then, the fat body tissues from 7‐day aged flies were dissected and measured by Raman or SRS imaging system.

For the starvation test, two groups of 2‐day *w^1118^* female adult flies (30 flies per group) were transferred to the 20% D_2_O‐labeled ST and HP food for 5 days labeling, respectively. Then, the labeled flies were starved on paper soaked with PBS. At 12 h, 24 h, and 36 h after starvation, 5 files from each group were sacrificed, and fat body was dissected and subjected to Raman measurements for the C‐D signal quantification.

To quantify LD size, the diameters of 50 LDs (larger than 4 μm that can be accurately measured) in 20 fat cells from 3 different individuals were measured by ImageJ software.

### 4.4 Fluorescent imaging of fatbody lipid droplets

Four files from each diet‐manipulated group were sacrificed, and fat bodies were dissected and fixed in 4% PFA (in 1 × PBS) for 15 min and then incubated in 1 µg/ml BODIPY™ 493/503 solution for 30 min. The stained tissues were imaged immediately by using two photon fluorescence microscopy at 800 nm.

To quantify LD size, the diameters of 20 LDs (larger than 4 μm that can be accurately measured) from 4 different individuals were measured by using ImageJ software.

### 4.5 Spontaneous Raman scattering microscopy

Raman spectra of all the tissue samples were measured by a Raman spectrometer connected to a confocal Raman microscope (XploRA PLUS, Horiba). A 532 nm diode line focus laser (~40 mW at the sample) was focused on the cells with the help of a 100× objective (MPLN100X, Olympus). The laser power on the sample was optimized so as to avoid any damage to the cells. A cooled charge‐coupled device (CCD) detector fitted to a 2400 grooves/mm grating spectrometer was used to detect the signal. Spectra were collected at 60 s acquisitions with an accumulation of 2. The background spectra were taken for each tissue point at the same focus plane and were subtracted from original spectrum immediately. Spectra were preprocessed using vector normalization and simplex normalized. Peaks were normalized to the phenylalanine peak at 1003 cm^−1^. The instrumental calibration was verified using the silicon line at 520 cm^−1^. The observed data were processed and analyzed using Prism software (Origin Lab Corporation, Northampton, MA).

### 4.6 Stimulated Raman scattering microscopy

An upright laser‐scanning microscope (DIY multiphoton, Olympus) with a 25× water objective (XLPLN, WMP2, 1.05 NA, Olympus) was applied for near‐IR throughput. Synchronized pulsed pump beam (tunable 720–990 nm wavelength, 5–6 ps pulse width, and 80 MHz repetition rate) and Stokes (wavelength at 1032 nm, 6 ps pulse width, and 80 MHz repetition rate) were supplied by a picoEmerald system (Applied Physics & Electronics) and coupled into the microscope. The pump and stoke beams were collected in transmission by a high NA oil condenser (1.4 NA). A high O.D. shortpass filter (950 nm, Thorlabs) was used that would completely block the Stokes beam and transmit the pump beam only onto a Si photodiode for detecting the stimulated Raman loss signal. The output current from the photodiode was terminated, filtered, and demodulated by a lock‐in amplifier at 20 MHz. The demodulated signal was fed into the FV3000 software module FV‐OSR (Olympus) to form image during laser scanning. All images obtained were 512 × 512 pixels, with a dwell time 80 μs and imaging speed of ~23 s per image. A background image was acquired at 2190 cm‐1 and subtracted from all SRS images using ImageJ.

### 4.7 Statistical analysis

Kaplan–Meier log‐rank test was performed for survival by using MATLAB (version 2021a). Others were tested by Student's *t* test, ANOVA with a post hoc Tukey's multiple comparison or ANOVA with a post hoc Dunnett's comparison test using GraphPad Prism software (version 7).

## CONFLICT OF INTEREST

The authors declare no competing interests.

## AUTHOR CONTRIBUTIONS

Lingyan Shi and Yajuan Li conceived the idea and designed the study. Yajuan Li conducted the experiments, analyzed the data, and performed statistical analyses with the help from Wenxu Zhang, Anthony A. Fung, and Lingyan Shi. Yajuan Li and Lingyan Shi wrote and revised the manuscript with the input from all other authors.

## Supporting information

Fig S1

Fig S2

Fig S3

Fig S4

Fig S5

Table S1

## Data Availability

The data that support the findings of this study are available from the corresponding author upon reasonable request.
