# Supplementary figures and images for: DO‐SRS imaging of diet regulated metabolic activities in Drosophila during aging processes
Source: Aging Cell. 2022 Mar 7;21(4):e13586. doi: 10.1111/acel.13586 (PMC9009230; doi:10.1111/acel.13586)

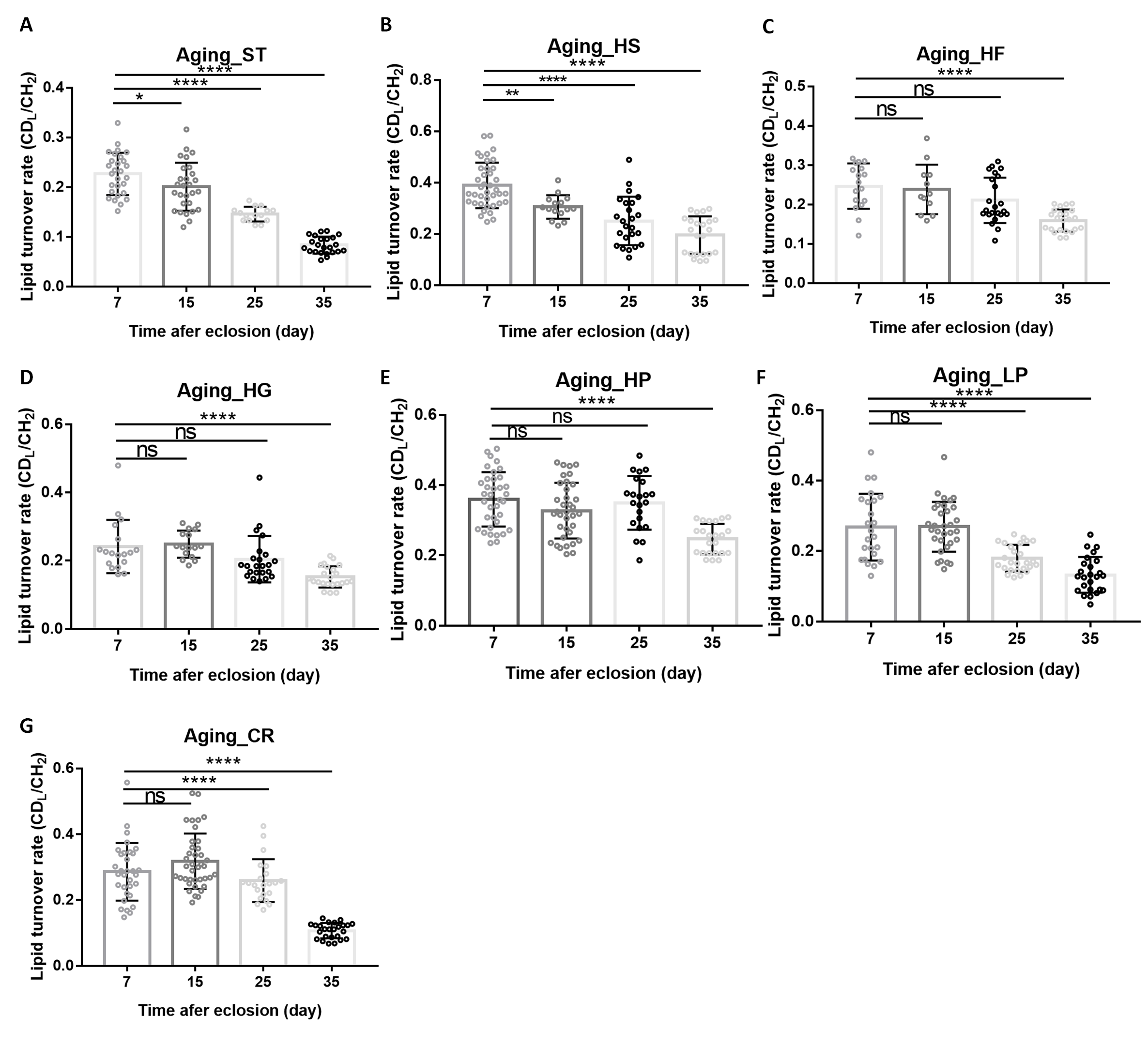

Supplement: Supplementary file 1 — Fig S1 [file ACEL-21-e13586-s006.tif]

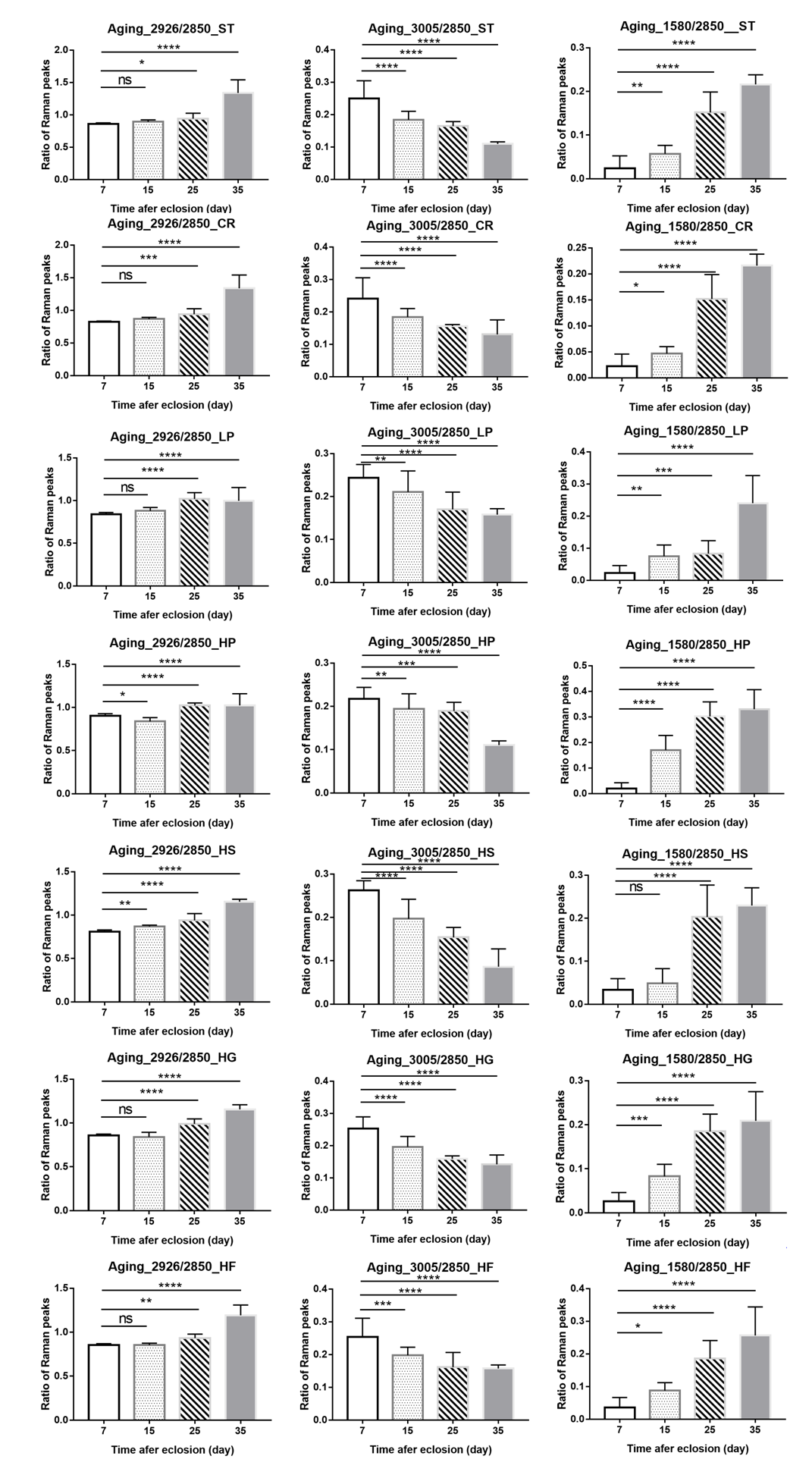

Supplement: Supplementary file 2 — Fig S2 [file ACEL-21-e13586-s003.tif]

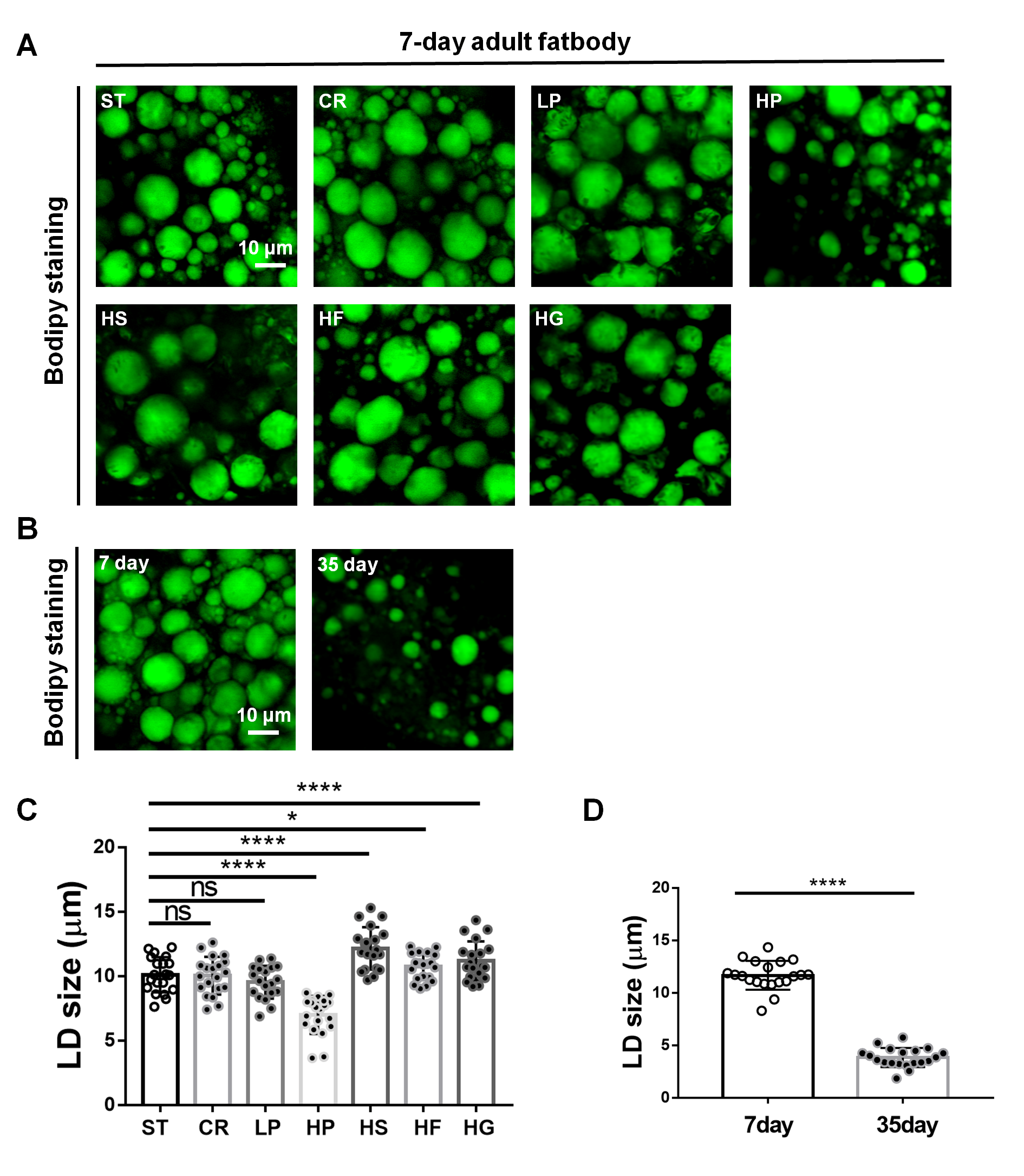

Supplement: Supplementary file 3 — Fig S3 [file ACEL-21-e13586-s002.tif]

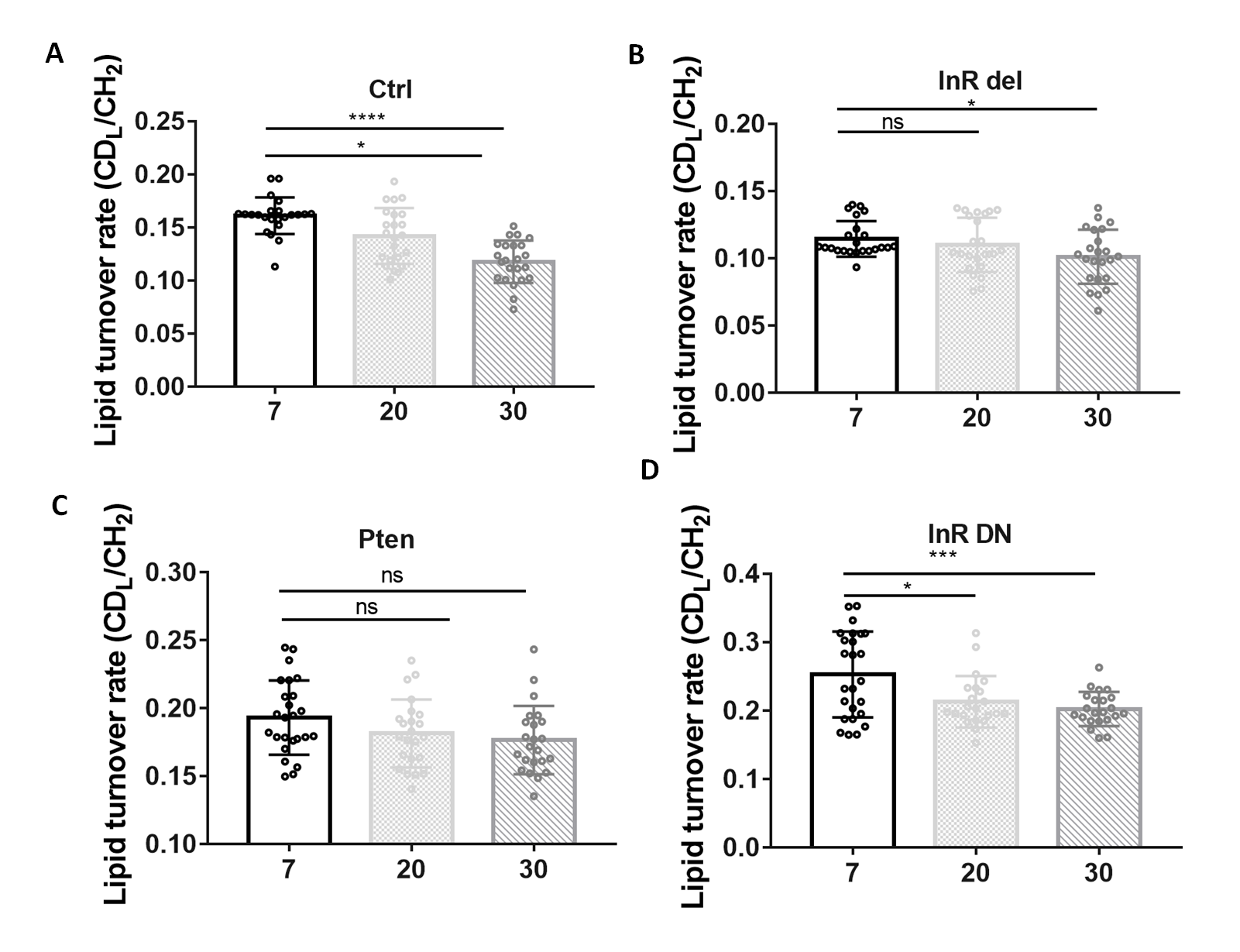

Supplement: Supplementary file 4 — Fig S4 [file ACEL-21-e13586-s004.tif]

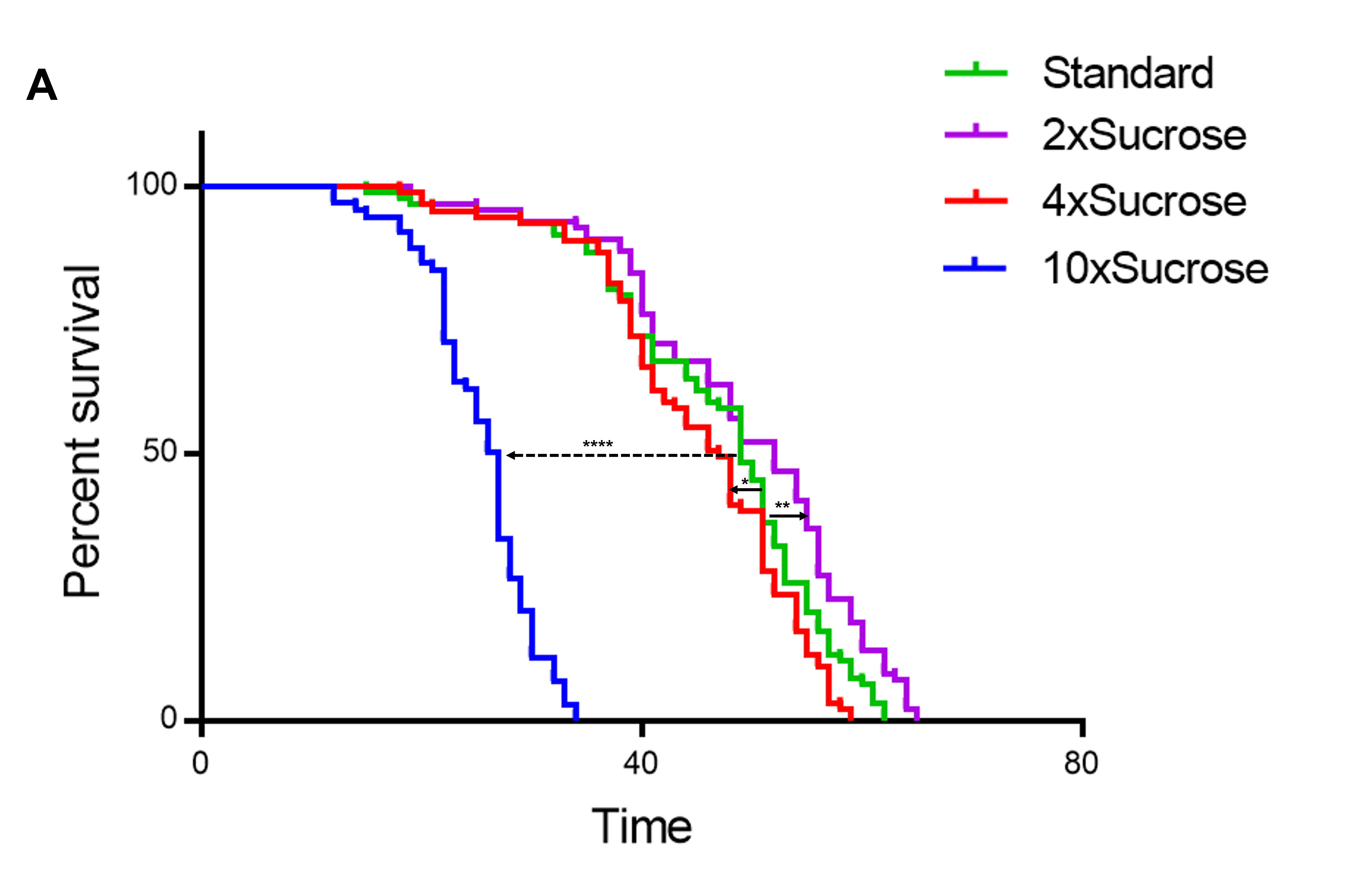

Supplement: Supplementary file 5 — Fig S5 [file ACEL-21-e13586-s001.tif]
