# Supplementary material for: DO‐SRS imaging of diet regulated metabolic activities in Drosophila during aging processes
Source: Aging Cell. 2022 Mar 7;21(4):e13586. doi: 10.1111/acel.13586 (PMC9009230; doi:10.1111/acel.13586)
Supplement: Supplementary file 6 — Table S1 [file ACEL-21-e13586-s005.docx]

**Tables**

Table S1. Food recipe for Drosophila.

| **per liter of water** | **Normal food** | **High sucrose** | **High glucose** | **High fructose** | **High protein** | **Low protein** | **Calorie restriction** |
| --- | --- | --- | --- | --- | --- | --- | --- |
| Yeast | 100g | 100g | 100g | 100g | 200g | 50g | 50g |
| Sucrose* | 50g | 100g | 100g | 100g | 50g | 50g | 25g |
| Cornmeal | 8g | 8g | 8g | 8g | 8g | 8g | 8g |
| Agar | 10g | 10g | 10g | 10g | 10g | 10g | 10g |
| Propionic acid (dissolved in 1L ddH_2_O) | 3g | 3g | 3g | 3g | 3g | 3g | 3g |
| Nipagin  (in ethanol) | 3g | 3g | 3g | 3g | 3g | 3g | 3g |

***** For high glucose and high fructose diets, sucrose was replaced with glucose and fructose, respectively.
